# Multisectoral, Combination HIV Prevention for Adolescent Girls and Young Women: A Qualitative Study of the DREAMS Implementation Trajectory in Zambia

**DOI:** 10.9745/GHSP-D-22-00089

**Published:** 2022-10-31

**Authors:** Joseph G. Rosen, Maurice Musheke, Drosin Mulenga, Edith S. Namukonda, Nrupa Jani, Michael T. Mbizvo, Julie Pulerwitz, Sanyukta Mathur

**Affiliations:** a Population Council, Lusaka, Zambia.; b Department of International Health, Johns Hopkins Bloomberg School of Public Health, Baltimore, MD, USA.; c Social and Behavioral Research, Population Council, Washington, DC, USA.

## Abstract

**Objective:**

To identify solutions to the implementation challenges with the DREAMS (Determined, Resilient, Empowered, AIDS-free, Mentored, and Safe women) Partnership in Zambia, this study examines the rollout and evolution of the DREAMS Partnership’s implementation.

**Methods:**

In September–October 2018, implementing partner (IP) staff (n=15) and adolescent girls and young women (AGYW) participating in DREAMS programming (n=32) completed in-depth interviews exploring early rollout and scale-up of DREAMS, experiences with program participation, and shifting service delivery approaches in response to emerging implementation challenges. Inductive and deductive thematic analysis of 47 interviews uncovered salient service delivery facilitators and barriers in the first 2 years of DREAMS implementation, which were subsequently mapped onto the following domains: reach, effectiveness, adoption, implementation, and maintenance.

**Results:**

Key implementation successes identified by IP staff included using standardized recruitment and risk assessment tools across IP organizations, using a mentor model for delivering program content to AGYW, and offering centralized service delivery at venues accessible to AGYW. Implementation challenges identified early in the DREAMS Partnership’s lifecycle were rectified through adaptive service delivery strategies. Monthly in-person coordination meetings were established to resolve IP staff jurisdictional disputes over recruitment and target setting. To address high participant attrition, IP staff adopted a cohort approach to sequentially recruit AGYW who enrolled together and provided social support to one another to sustain involvement in DREAMS programming. Prominent barriers to implementation fidelity included challenges recruiting the highest-risk AGYW (e.g., those out of school), limited resources to incentivize participation by young women, and inadequate planning to facilitate absorption of individual DREAMS interventions by the public sector upon project conclusion.

**Conclusions:**

Delivering multisectoral HIV prevention programs like DREAMS with fidelity requires a robust implementation infrastructure (e.g., adaptable workplans and harmonized record management systems), early coordination between IP organizations, and sustained financial commitments from donors.

## Abstract (precis)

Our study of DREAMS implementation in Zambia identified key implementation successes and challenges experienced by implementing partners and program participants, from program rollout and throughout its evolution.

## Teaser

**Key Findings:**

- The DREAMS Partnership is a combination HIV prevention approach in sub-Saharan Africa to reduce risk in adolescent girls and young women (AGYW), but its multisectoral approach poses challenges for implementing partners.
- Comprehensive coordination systems between implementing partner organizations are needed in multisectoral programming to build the necessary infrastructure for effective implementation.
- Securing financial commitments, for both primary interventions directed toward AGYW and secondary interventions directed toward their male partners and parents, is important for engaging these stakeholders and addressing structural drivers of HIV risk among AGYW.
- Real-time adaptation of implementation strategies helped address challenges with recruiting AGYW with the highest risk and retaining AGYW in the program.

**Key Implication:**

- To administer DREAMS sustainably and effectively, implementing partners must invest in robust implementation infrastructure, including harmonized electronic data capture systems and flexible workplans, requisite for multisectoral HIV prevention programming.

## INTRODUCTION

Four decades into the HIV epidemic, new infections among adolescent girls and young women (AGYW) remain persistently high. Sixty percent of new HIV diagnoses in adolescents and young people (aged 15–24 years) are in AGYW— a rate equivalent to 1,000 new infections daily.^1^ A majority (80%) of these incident cases occur in sub-Saharan Africa.^1^ As throughout East and Southern Africa, the gendered dynamics of HIV transmission in Zambia, with an annual HIV incidence in AGYW that is 13 times higher than in similarly aged men,^2^ demand holistic prevention approaches tackling HIV vulnerabilities among AGYW. Out-of-school girls and women, accounting for nearly 60% of school-aged AGYW in Zambia,^3^ are more likely to acquire HIV than in-school AGYW.^4^^,^^5^ Experiences with violence can likewise attenuate AGYW’s capacity to protect themselves from HIV infection.^6^^–^^9^ Uptake of HIV services, from counseling and testing to treatment, by male partners of AGYW also remains suboptimal, exacerbating these aforementioned challenges experienced by AGYW.^10^^–^^12^ To successfully reduce HIV incidence among AGYW, prevention strategies must look beyond conventional biomedical approaches and meaningfully address the social and structural dimensions of AGYW’s HIV risk.^13^

In response to these persistent HIV burdens in AGYW, the U.S. President’s Emergency Plan for AIDS Relief (PEPFAR) launched the DREAMS Partnership in 2014. DREAMS supports the development of Determined, Resilient, Empowered, AIDS-free, Mentored, and Safe women by delivering a set of interventions addressing multilevel drivers of HIV risk in AGYW (e.g., school attrition, unemployment, intimate partner violence, and unmet need for preventive health commodities).^14^ With a primary goal of empowering AGYW to reduce their risk of HIV infection, the core package of interventions is differentiated to specific target audiences—from direct program participants (i.e., AGYW) to proximal and distal actors shaping the HIV risk environment for AGYW (i.e., male partners and parents)—to achieve 3 objectives: strengthen families, mobilize communities for change, and reduce risk of sexual partners.^14^

The DREAMS Partnership offers valuable learning opportunities through the novel, multisectoral approach to implementation of a combination of HIV prevention interventions that have historically been delivered in a siloed fashion. When delivered in combination, individual interventions included in the DREAMS core package are more accessible to AGYW, customizable and tailored to individual client’s needs, and positioned to synergistically address overlapping drivers of HIV risk.^15^ Combination service delivery models like DREAMS also offer promising returns on investments—by packaging individual interventions into a streamlined service delivery platform, they can magnify the expected benefits of these same interventions delivered independently, optimizing cost-effectiveness.^16^^–^^18^

Nevertheless, relative to standalone HIV initiatives, combination HIV prevention programs like DREAMS require innovation, flexibility, and integration—characteristics that pose unprecedented challenges to intervention fidelity and sustainability. Given the unique service delivery and implementation context in which DREAMS is situated, implementation science research offers a useful paradigm for examining the implementation trajectory of complex initiatives like DREAMS. Unlike traditional process or impact evaluation approaches, implementation science focuses more on the lived experiences of implementation and less on the outputs or outcomes of implementation. By disentangling the mechanics of program implementation from their measured impacts, implementation science addresses how interventions work to better understand how they achieve or fail to achieve their stated objectives.^19^

Relative to standalone HIV initiatives, combination HIV prevention programs like DREAMS require innovation, flexibility, and integration—characteristics that pose unprecedented challenges to intervention fidelity and sustainability.

This qualitative study examines the DREAMS implementation trajectory in Zambia by eliciting AGYW and implementing partner (IP) staff perspectives and experiences with various dimensions of the DREAMS Partnership’s implementation chronology, specifically: identifying and reaching appropriate target audiences; delivering the appropriate content and services to these audiences; adapting/modifying service delivery to address emergent challenges; retaining participants in primary and secondary interventions over time; and facilitating absorption of individual interventions by the public sector.

### DREAMS Rollout and Scale-Up in Zambia

DREAMS includes individual (primary and secondary service packages) (Table 1) and contextual interventions delivered in 15 countries that account for half of new HIV infections among AGYW worldwide. The primary service package in Zambia initially consisted of a social-asset-building curriculum (i.e., age-appropriate “safe space” sessions focused on HIV prevention-related topics), condom promotion and distribution, HIV testing services, combination socioeconomic support (i.e., financial literacy, training, and education support), and school-based HIV and violence prevention education. Secondary interventions for eligible AGYW include adolescent-friendly family planning services, preexposure prophylaxis (PrEP) for HIV prevention, postviolence care, education subsidies, and parenting/caregiver programs. Contextual interventions include community-based services that cannot necessarily be delivered discretely to DREAMS participants but can be prioritized in specific communities (e.g., with higher HIV burdens) or tailored to specific populations (e.g., male partners).^15^ In Zambia, contextual interventions included community mobilization and norms-changing activities focused on violence prevention and gender equity.

**TABLE 1.** Primary and Secondary Individual DREAMS Interventions for Adolescent Girls and Young Women in Zambia

	**Individual Interventions**	**Description**
Primary	Social-asset building	13 age-appropriate Safe Space sessions focused on HIV prevention–related topics (e.g., puberty, consent) delivered in community-based “hubs”
	Condom promotion and distribution	Education, promotion, and availability of condoms through Safe Spaces and adolescent health services
	HIV testing services	HIV testing and linkage to services (e.g., HIV care and treatment, other DREAMS services)
	Combination socioeconomic support	Cash transfers Village and loan savings groups Financial literacy education
	School-based HIV and violence prevention	Education on HIV and gender-based violence for in-school AGYW (ages 15–19 years only)
Secondary	Adolescent-friendly family planning services	Expanded access to contraception (i.e., long-acting reversible methods) and other reproductive health services
	PrEP	Education and linkage to PrEP for at-risk women
	Postviolence care	Screening and linkage to postviolence services, including PEP
	Education subsidies	Money to support educational expenses, including school fees, uniforms, and transportation
	Parenting and caregiver programs	Family strengthening programming, including HIV risk and violence prevention awareness for parents of AGYW (i.e., Families Matter!)

Abbreviations: AGYW, adolescent girls and young women; DREAMS, Determined, Resilient, Empowered, AIDS-free, Mentored, and Safe women; PEP, post-exposure prophylaxis; PrEP, preexposure prophylaxis.

The combination, or “layers,” of DREAMS interventions delivered to AGYW are determined by client needs and assessments of an AGYW’s HIV vulnerability.^14^ A hallmark of DREAMS, “layering” is a client-centered approach involving the assessment of AGYW’s HIV risk to determine the appropriate combination of services that should be offered to them. Risk and vulnerability factors used in determining program eligibility and the combination of DREAMS services for AGYW include multiple sexual partnerships, inconsistent or no condom use during sex, transactional sex, history of sexually transmitted infections, experiences with violence, substance use, out-of-school status, and orphanhood. IP organizations delivering individual DREAMS interventions are integrated primarily through formal referral mechanisms, whereby implementing staff must actively link (through accompaniment or individual case management) AGYW to services offered by other IP organizations within the DREAMS Partnership.^15^ Individual interventions included in the primary service package are also delivered through community-based platforms called DREAMS centers or venues located within communities where AGYW congregate and participate in DREAMS-sponsored activities and services.

DREAMS programming was first introduced in Zambia in 2016, with services initially implemented in 3 districts with high HIV burdens among AGYW: Chingola, Lusaka, and Ndola.^20^ In 2018, DREAMS programming was expanded to 5 additional districts: Chipata, Kabwe, Kapiri, Kitwe, and Livingstone. Sites for DREAMS implementation were selected by PEPFAR, in consultation with government line ministries, based on background HIV transmission dynamics.^8^^,^^14^ In 2020, PEPFAR expanded DREAMS implementation to 6 additional districts (Mongu, Monze, Mazabuka, Kasama, Luanshya, and Mufulira), based on evolving HIV epidemic profiles in these subnational units.^20^ With more than US$85 million in investments from PEPFAR and multilateral partners to date, by 2021, over 3,300 PrEP initiations among AGYW were documented in subnational units with ongoing DREAMS implementation.^21^ More than 800,000 AGYW in Zambia received at least some component(s) of the DREAMS primary service package by 2022.^21^ Studies have also attributed increased HIV testing coverage and reductions in sexual violence victimization among AGYW to DREAMS implementation in Zambia.^22^

## METHODS

### Study Setting

The present study is nested in a larger portfolio of implementation science research of the DREAMS Partnership in 7 countries.^23^ In Zambia, a longitudinal mixed methods study was proposed to (1) measure uptake and sustained participation in combination HIV prevention activities offered through DREAMS (prospective cohort study) and (2) document program participant and IP staff perspectives of and experiences with program participation and service delivery (qualitative in-depth interviews). Findings from the parent study’s quantitative component have been published elsewhere.^8^^,^^22^^,^^24^^,^^25^

This study was conducted in 2 urban districts with ongoing DREAMS implementation: Lusaka (Lusaka Province) and Ndola (Copperbelt Province). AGYW in these districts are disproportionately affected by numerous health and development challenges, including high HIV incidence, adolescent pregnancy, and school attrition. Women’s HIV prevalence estimates for both provinces are among the highest in Zambia (Copperbelt: 17.3%, Lusaka: 17.9%).^2^ Additionally, the proportion of teenagers who have begun childbearing remains high (Copperbelt: 21.0%, Lusaka: 14.9%), despite increased provision of comprehensive sexuality education in schools and expanded access to modern contraceptive methods.^3^^,^^26^

The study was conducted in 2 urban districts in 2 provinces in Zambia with high HIV prevalence and high proportion of teenagers who have begun childbearing.

### Study Populations

This study included 2 populations: (1) AGYW who participated in DREAMS and (2) IP staff members. As an AGYW’s age determines the type of services for which she is eligible,^14^ girls and women across 3 DREAMS priority age groups (10–14 years, 15–19 years, and 20–24 years) were included in the study. Eligible AGYW included those currently participating in DREAMS programming, who graduated from DREAMS (i.e., received a certificate acknowledging completion of 13 safe space sessions), or withdrew before certificate conferral.

IP staff included site-level (involved with direct service delivery) and management (overseeing DREAMS implementation) personnel. Site-level staff consisted of mentors (who facilitate safe space sessions and provide psychosocial support and counseling to AGYW), connectors (who administer risk assessment screenings to AGYW and facilitate referrals to health services in the public sector), and site coordinators. Management staff included program managers, monitoring and evaluation specialists, technical advisors, and other senior leadership (e.g., program directors and chiefs of party) from nongovernmental organizations contracted to implement individual DREAMS interventions.

### Research Conceptual Framework: RE-AIM

The RE-AIM Framework conceptualizes the public health impact of an intervention as a product of the interaction between 5 factors: (1) reach (intervention coverage), (2) effectiveness (achievement of expected/desired outcomes), (3) adoption (acceptability and uptake of intervention), (4) implementation (intervention effectiveness or adherence to service delivery strategies like layering), and (5) maintenance (sustainability of program impact and implementation).^27^ RE-AIM helpfully assesses interventions that address multiple overlapping causes and holistic systems,^28^ including combination HIV prevention programs like DREAMS. As illustrated in the Figure, the RE-AIM framework was used to formulate research questions that guided data collection and analysis for the present study.

**FIGURE.**
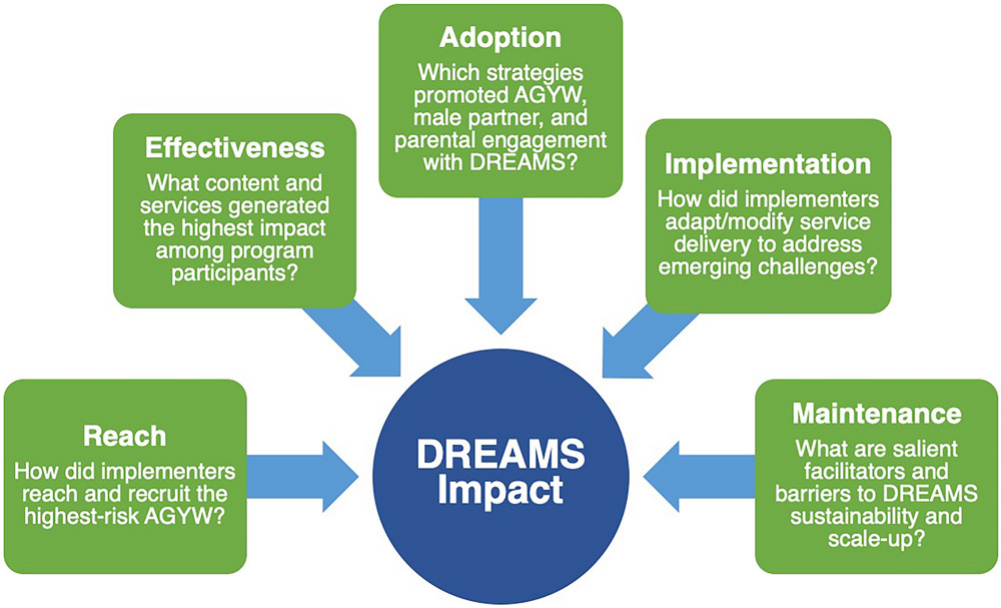
Adapted RE-AIM Framework for DREAMS Implementation Science Research in Zambia Abbreviations: AGYW, adolescents and young women; DREAMS, Determined, Resilient, Empowered, AIDS-free, Mentored, and Safe women.

### Recruitment and Data Collection

Between September and October 2018 (approximately 2 years after DREAMS was introduced in Zambia), AGYW were purposively recruited through DREAMS participant registries with the assistance of site-level staff. AGYW and site-level staff were approached about study participation in DREAMS centers. A purposive sample of AGYW, stratified by DREAMS completion status (i.e., received a certificate of completion or withdrew before certificate conferral) and district (i.e., Lusaka and Ndola), facilitated the inclusion of AGYW across program exposure experiences and implementation settings. Management staff were recruited during monthly DREAMS coordination meetings in Lusaka, and snowball sampling was used to identify other program staff for recruitment based on recommendations from participating management staff.

Semistructured in-depth interviews, lasting 30–60 minutes, were conducted by experienced qualitative research assistants in English and/or combination of Bemba and Nyanja. Topics addressed in AGYW interviews included: experiences with DREAMS recruitment modalities; motivations for DREAMS participation; perspectives on individual interventions offered through DREAMS; barriers to program participation and sustained engagement; and perceived impact of DREAMS on AGYW, their male partners, and their communities. IP staff interviews focused primarily on early challenges coordinating DREAMS implementation, administrative and logistical successes and shortcomings, strategies adopted to mitigate emerging implementation challenges, and perceived sustainability of the DREAMS service package.

### Data Analysis

Interviews were audio-recorded, transcribed verbatim, and—when required—translated into English. Thematic analysis of interview transcripts was conducted by 5 study staff using a hybrid inductive-deductive analytic approach, applying the tenets of multicycle coding and the Framework Method.^29^^,^^30^ In the first cycle of coding, study staff read each interview closely and generated a list of codes representing themes emerging from transcripts. Next, analysts grouped themes identified during first-cycle coding into discrete overarching categories. The emerging categories identified from the second-coding cycle guided the development of data synthesis templates, which were differentiated to AGYW and IP staff interviews, respectively. These templates facilitated the abstraction of textual data from interview transcripts and were structured using overarching research questions presented in the study-adapted RE-AIM framework (Figure).

Following additional close readings of transcripts, analysts populated 1 template per transcript. Each populated data synthesis form contained abstracted textual data (i.e., quotes) and corresponding summaries and interpretations of the data. Analysts and study investigators convened regularly to discuss populated templates, describe salient themes, and identify thematic patterns. Once all templates were populated with textual data, matrices of coded text segments were assembled collaboratively among analysts to further condense and synthesize textual data. These matrices helped identify thematic patterns across participant subgroups and confirmed the salience of emerging themes. Salient themes were then collated across AGYW and IP staff interview transcripts and mapped onto RE-AIM framework domains.

### Ethical Approval

The study was approved by the Population Council Institutional Review Board (New York, NY, USA) and ERES Converge (Lusaka, Zambia). AGYW and IP staff aged 18 years and older provided written informed consent before participation. Adult caregiver written consent and informed assent were required and obtained for AGYW aged younger than 18 years.

## RESULTS

Table 2 summarizes the characteristics of interviewed AGYW (n=32), stratified by district. The mean age was 16 years (standard deviation: 4.5 years). Half of AGYW were aged 10–14 years and completed DREAMS programming. Most were unmarried (97%), nulliparous (97%), and in school (78%). When restricted to AGYW aged 15–24 years (n=16), 6 (36%) were out of school. Among IP staff (n=15), 8 site-level and 7 management staff were included (Table 3). Most interviewed IP staff members were based in Lusaka (73%) and oversaw social-asset–building (i.e., safe spaces) implementation for the DREAMS Partnership (56%).

**TABLE 2.** Characteristics of Interviewed AGYW Who Completed or Withdrew From DREAMS Programming in Zambia, by District

	District		Total, No. (%) (N=32)
	Lusaka (n=16)	Ndola (n=16)	
Mean age, in years (SD)	17	15	16 (4.5)
Age group			
10–14 years	8	8	16 (50.0)
15–19 years	2	7	9 (28.1)
20–24 years	6	1	7 (21.9)
Marital status			
Unmarried	16	15	31 (96.9)
Married or cohabiting	—	1	1 (3.1)
Parity			
Nulliparous	15	16	31 (96.9)
>1 child	1	—	1 (3.1)
Education			
In-school	13	12	25 (78.1)
Out-of-school	3	4	7 (21.9)
DREAMS completion status			
Completed	8	8	16 (50.0)
Withdrew	8	8	16 (50.0)

Abbreviations: DREAMS, Determined, Resilient, Empowered, AIDS-free, Mentored, and Safe women; SD, standard deviation.

**TABLE 3.** Characteristics of Interviewed DREAMS Implementing Partner Staff in Zambia, by District

	**District, No.**		**Total (N=15)**
	**Lusaka (n=11)**	**Ndola (n=4)**	
Staff cadre			
Management	7	—	7
Site level	4	4	8
Job function			
Senior leadership	2	—	2
Technical advisor	1	—	1
Program manager	4	—	4
Site coordinator	1	—	1
Connector	1	1	2
Mentor	2	3	5
DREAMS program area			
Social asset building	5	4	9
Combination socioeconomic support	1	—	1
School-based HIV and violence prevention	1	—	1
Preexposure prophylaxis	1	—	1
Postviolence care	1	—	1
Education subsidies	1	—	1
Parenting and caregiver programs	1	—	1

Abbreviations: DREAMS, Determined, Resilient, Empowered, AIDS-free, Mentored, and Safe women.

Emerging themes and insights from AGYW and IP staff interviews are presented according to their corresponding domains from the RE-AIM framework: reach, effectiveness, adoption, implementation, and maintenance.

### Reach: Recruiting the Highest-Risk AGYW

IP staff communicated numerous strategies that were used to identify eligible AGYW for participation in DREAMS. One key recruitment strategy was a standardized risk assessment tool, deployed across IP organizations and service delivery settings. The tool contained questions eliciting AGYW’s household characteristics, financial and schooling status, and sexual risks—all of which were collated to identify the appropriate combination of DREAMS interventions that should be offered to newly enrolled AGYW. For many IP staff members, the tool effectively harmonized indicators of “HIV vulnerability” across the various IP organizations implementing the DREAMS core service package. For many IP staff, the screening tool harmonized HIV risk typologies, enabling different IP staff to recruit AGYW using streamlined definitions and heuristics for HIV vulnerability measurement:

*One girl should not receive (only) 1 service. … There is tracking done … to ensure that only the most vulnerable are supported. … Not everyone gets the same services. For instance, the 10–14[-year-olds], probably few of them would receive family planning … but they would be given information about the need … to save money*. —Program manager, social-asset building, Lusaka

A key recruitment strategy was using a standardized risk assessment tool to identify the appropriate combination of DREAMS interventions that should be offered to newly enrolled AGYW.

In some cases, however, the highly sensitive questions contained in the screening tool discouraged AGYW from disclosing specific experiences or behaviors that would otherwise render them eligible for more DREAMS interventions. This is when IP staff relied heavily on DREAMS mentors and other site-level staff to adapt implementation strategies, including delaying the use of risk assessments until after having 3–4 encounters with AGYW.

*It was supposed to be administered the first time … you are in contact with the girl, but we realized that the questions were a bit more detailed and sensitive. . . . Most of the girls used to run away from certain questions that were in the screening form. . . . Instead of asking the girls the questions at the beginning, it is better to give them a period of time … and then when you see that they have started opening up, that’s when you introduce the screening form.* —Site coordinator, social-asset building, Lusaka

To enroll AGYW aged 10–24 years, IP staff recruited AGYW from venues where they could be easily located, specifically schools. Some management staff suggested this approach likely excluded other vulnerable groups of AGYW (i.e., out-of-school girls) from learning about and participating in DREAMS.

*As much as we would like to recruit [AGYW] from hotspots, bars, health facilities, and door-to-door, the easiest point of recruitment is schools because we know they are there.* —Senior leadership, social-asset building, Lusaka

Indeed, most interviewed AGYW reported first learning about DREAMS from outreach activities at their schools.

*They came to our school and registered us. They told us to come to the [DREAMS] center. That is how we started participating.* —AGYW, age 17 years, Lusaka

### Effectiveness: Developing Content, Services, and Systems that Maximize Program Impact

AGYW and IP staff shared mixed perspectives on whether DREAMS programming achieved its intended impacts. When reflecting on the program’s impacts on their livelihoods, some AGYW spoke of heightened awareness of their HIV risk and shifts in behavior to mitigate this risk, including reducing their number of sexual partners and encouraging their partners to get tested for HIV. Others, particularly younger girls, explained how DREAMS equipped them with the information, life skills, and self-efficacy to confront harmful gender norms that perpetuated violence and HIV transmission in their communities. IP staff shared similar observations of improved health care access and positive shifts in health behavior, describing increased HIV testing, heightened demand for modern contraception, and reductions in adolescent pregnancies—all of which they attributed at least partially to DREAMS programming.

*Before DREAMS, I didn’t know anything. . . . Boys used to touch my breasts, so when I learned that they shouldn’t, I stopped them.* —AGYW, age 11 years, Lusaka

*We take the service to where they [AGYW] are found. . . . We make arrangements with a nurse. . . . Then, they go and access family planning services. . . . Now information has been preached to them. They are very happy and comfortable with the services we are offering.* —Connector, social-asset building, Ndola

AGYW and IP staff shared mixed perspectives on whether DREAMS programming achieved its intended impacts.

Simultaneously, AGYW and IP staff highlighted specific program components failing to achieve desired impacts. AGYW participating in combination socioeconomic support interventions, for instance, explained that these programs did not initially provide the material resources (i.e., loans and start-up capital) required for savings/lending groups and microenterprises. Business start-up kits have since been folded into DREAMS socioeconomic support interventions.

*The beginning was challenging because that is when you start saving. Some were having shortfalls because it was the first time. . . . When they borrow, that is when money would become active, not when a person has just joined. There were challenges because she hadn’t started earning money.* —AGYW, age 24 years, Ndola

*[AGYW] say they do not have money, meaning … their spouses can’t give them money or extra income to invest.* —Site coordinator, social-asset building, Lusaka

Likewise, IP staff identified challenges with condom promotion messaging, which they suggested was insufficient for capacitating AGYW, especially married young women, to negotiate condom use with male partners.

*I still feel like risk perception is low. . . . When you interact with these girls, you notice that they don’t have that fear. . . . The fear is just not there. A lot of them are still mentioning … that they can’t negotiate condom use.* —Program manager, social-asset building, Lusaka

### Adoption: Incentivizing AGYW, Male Partner, and Parental “Buy In” to DREAMS

Age-appropriate services delivered in safe environments (DREAMS centers) were required to incentivize participation among different age groups. These centers serve as launch points for DREAMS services, including the safe space groups. AGYW, especially younger girls, embraced the DREAMS centers because they felt welcome and comfortable discussing salient issues regarding sex and sexuality. Additionally, these venues offer accessible spaces in AGYW’s communities to engage meaningfully with similarly aged girls and safely share perspectives on sensitive topics.

*I feel good when I come. They [DREAMS mentors] teach us…I don’t have friends, so here is where I have friends.* —AGYW, age 17 years, Ndola

AGYW also spoke highly of DREAMS mentors, who are the backbone of site-level program implementation. Mentors—all of whom are women—are responsible for a range of activities, including recruitment, outreach, facilitation, and data management/entry. Some interviewed mentors described duties that far exceeded their formal employment obligations, like paying out-of-pocket for AGYW’s expenses. Many AGYW attributed their continuation in DREAMS to the trusting relationships they forged with their mentors. In turn, mentors also reflected on how they derive motivation from their work, which they describe as highly valued and respected by AGYW.

*I’m looked up to as a role model in the community. . . . I have learned a lot from the girls that I have been mentoring. . . . I never thought I would change these girls’ lives or the way they think. . . . All this makes me happy and walk with my head up in the community.* —Mentor, social-asset building, Lusaka

AGYW spoke highly of DREAMS mentors, who are the backbone of site-level program implementation and responsible for recruitment, outreach, facilitation, and data management.

IP staff shared that program engagement among younger AGYW proved easier than for older AGYW because of heterogeneous motivations for enrollment and perceived benefits of DREAMS participation. Whereas younger AGYW were enticed by community-building and educational activities, older AGYW were motivated by economic support and training opportunities. Among site-level staff, the perceived lack of immediate financial or material benefits to participating in primary DREAMS interventions, like safe spaces, drove high discontinuation rates among older AGYW.

*The 20- to 24-year-olds—their expectations are high. For them, it is either you are taking them to school, or you want to find out what business they want to do, then you provide them with money.* —Mentor, social-asset building, Lusaka

While AGYW overwhelmingly suggested their parents and partners were supportive of—and, in some cases, encouraged—their participation in DREAMS, IP staff reported fewer successes with direct male partner and parent engagement. Efforts to recruit AGYW’s fathers into caregiving interventions, for example, were constrained by implementation that was inattentive to competing priorities (i.e., work schedules) and limited financial support for mobilization and sensitization. One such caregiving intervention, Families Matter!, struggled to recruit fathers of AGYW.

*Enrollment for fathers is at about 2% of the total enrollment of the program. . . . The mothers are more available during the day. . . . Some of them [fathers] were talking about when the mother comes home, they tell him what they learned, but I think it is just more of … the perception of such programs for men. . . . I have gotten the impression that they [fathers] aren’t really interested.* —Program manager, parenting and caregiver programs, Lusaka

Some IP staff explained that DREAMS contracts supported mobilization activities for AGYW only and could not explicitly finance outreach to other program audiences, like partners or parents. In these circumstances, IP staff mobilized external resources to subsidize these vital recruitment efforts or were left unable to mobilize, sensitize, or recruit altogether.

Some IP staff explained that DREAMS contracts supported mobilization activities for AGYW only and could not explicitly finance outreach to other program audiences, like partners or parents.

### Implementation: Designing Adaptive and Responsive Systems to Address Emerging Challenges

To implement effectively, the DREAMS Partnership required new systems of communication, coordination, and management across a consortium of IP organizations responsible for different, albeit complementary, components of the DREAMS core service package. Management staff reported that project coordination, including harmonizing monitoring and evaluation systems, within the IP consortium was among the most prominent challenges early in the DREAMS implementation trajectory. While some IP organizations had existing project infrastructure that could be reconfigured to accommodate new DREAMS workplans, other IP organizations with newly awarded DREAMS contracts were expected to meet ambitious program targets with neither preexisting infrastructure nor well-defined coordination systems between IP organizations to implement successfully. Numerous management staff explained how this initially fomented competition and tension between IP organizations.

*All of us [IP organizations] were going to mobilize our own girls. . . . We found that organizations started clashing. We are using 1 project for mobilization. Others are using safe spaces for mobilization. You will find that … others mobilized the same girls that you have already mobilized.* —Program manager, education subsidies, Lusaka

Incongruent record management systems additionally stymied efforts to monitor individual interventions delivered to DREAMS participants, particularly in an implementation ecosystem with different partners. Management staff especially advocated for more robust electronic data capture systems, which could be harmonized across IP organizations to effectively track service combinations.

*Being able to capture all the services the girl is receiving from different service delivery points, be it the Ministry of Health facility or another newly funded partner, still remains a challenge … Not all girls really take the referral forms or, if they do, it doesn’t find its way back for you to know that they actually went and got that service.* —Senior leadership, social-asset building, Lusaka

To address these coordination challenges, IP organizations established monthly DREAMS coordination meetings, where management staff from different partner organizations convened in person to discuss ongoing recruitment activities, review service delivery workplans, and troubleshoot implementation challenges. These meetings helped resolve implementation disputes within the consortium and fostered collaboration among IP organizations.

*At the beginning, everyone was trying to figure out how you put the pieces together. Everyone was running with their own targets. . . . There were different partners implementing DREAMS, so sometimes the schools were confused. . . . I haven’t heard recently reports of volunteers clashing or where different partners are looking for the same girl.* —Senior leadership, social-asset building, Lusaka

Another significant implementation challenge that emerged early in the DREAMS implementation lifecycle was AGYW attrition. In response, IP staff pivoted recruitment strategies and began enrolling AGYW into DREAMS using a cohort-based approach. Cohorts initiated and attended 13 safe space sessions before receiving certificates of completion, after which new cohorts could be enrolled into DREAMS. This approach ensured a manageable volume of AGYW received DREAMS programming simultaneously, which facilitated more effective program management (i.e., ensuring AGYW received combinations of services that addressed their individual needs) and continuity of service delivery. Nonetheless, this approach limited the number of AGYW that could participate in DREAMS, creating additional barriers to program participation.

*There are many girls who want to participate, but if you look at our model, we have a certain number we can serve in any given year … If each girl has to go through 13 sessions and can only meet over the weekend once or twice, and 1 mentor can only have 3 to 4 groups, then there is a maximum number you can serve in a year.* —Senior leadership, social-asset building, Lusaka

### Maintenance: Sustaining Service Delivery and Program Impact

The DREAMS Partnership developed a service delivery model in close collaboration with government line ministries, with a vision of transitioning oversight of individual interventions to the public sector once IP organizations demonstrated specific services could be implemented with fidelity. While IP staff offered numerous anecdotes of successful program implementation and target achievement within their respective DREAMS portfolios, management staff identified the DREAMS model’s resource intensiveness (e.g., personnel, training inputs) as a potential transition planning bottleneck. Line ministries would be responsible for not only integrating DREAMS programming into their existing menu of services but also shouldering administrative overhead and other program-related expenses, especially if donor investments were to dwindle in the future.

*The DREAMS model is not a cheap model. You need mentors. . . . You have to invest in training. The initial training is 10 days, but you also have to continue retraining them and … you have to compensate at least for the lost time and ensuring that these mentors go from house to house and continue talking to the girls, which means you have to bring in a supervisory system to ensure that actually happens*. —Senior leadership, social-asset building, Lusaka

Given the constellation of competing priorities that line ministries must weigh in their financial planning, a successful transition would require sustained investments in personnel and resources, as well as transition strategies that are integrated into IP workplans early in the implementation lifecycle.

At the site level, IP staff reflected on how absorption of DREAMS activities by the public sector would require a strong fiscal commitment to transition planning and service scale-up. For example, one IP described the burden DREAMS imposed on the health system, which was responding to increased demand for health services among AGYW without additional personnel or resources.

*We have seen an increase in the workload because we are attending to more … young people, which has never been the case, so they [health providers] started advocating for stipends and allowances.* —Program manager, school-based HIV and violence prevention, Lusaka

Management staff also described costs and sustainable financing of the DREAMS mentor model as salient challenges to program sustainability. In addition to overseeing and implementing programming for up to 30 AGYW at one time, mentors and other site-level staff, though presently salaried employees, were initially expected to work as volunteers, receiving only small stipends to offset transportation costs and other small expenses. IP staff, including mentors themselves, noted how these circumstances created stressful working conditions, resulting in high turnover and requisite supplementary resources to rehire and retrain staff.

*Facilitators should not run more than 6 groups per week. There can be a lot of burnout, and they can’t deliver as expected. . . . Keeping them motivated … will mean having their facilitation money and mobilization money raised in good time.* —Program manager, parent and caregiving programs, Lusaka

## DISCUSSION

A key finding of this qualitative study was the necessary leadership of site-level staff (i.e., mentors, connectors, and site coordinators) in real-time modification of DREAMS implementation strategies. These staff not only played key roles in AGYW recruitment and day-to-day operations but were also critical to adapting DREAMS service delivery approaches, like timing of screening tool administration for AGYW. While these staff were highly motivated, revered by DREAMS participants, and completed duties far exceeding their contractual obligations, they were initially undercompensated for their labor, working for little to no pay. The high staff turnover described by IP staff can be attributed, in part, to poor compensation and excess psychosocial demands. Based on past experiences in HIV and other sexual and reproductive health programs,^31^^–^^33^ reconfiguring program finances to fairly compensate site-level staff, including mentors and connectors, is essential for preventing turnover and modeling gender-equitable wage policies, given that all site-level staff were women. Provision of psychosocial support and generous health-related leave time can also help mitigate emotional strain and prevent burnout.

Reconfiguring program finances to fairly compensate site-level staff, including mentors and connectors, is essential for preventing turnover.

Another key challenge gleaned from IP staff interviews was perceived donor expectations to demonstrate achievement of ambitious recruitment and service delivery targets in the absence of comprehensive coordination systems between IP organizations. Compared to other combination HIV prevention programs implemented in East and Southern Africa,^34^^–^^36^ the DREAMS Partnership in Zambia awarded contracts to various subpartners to implement individual interventions, instead of delegating implementation to a single IP organization within a geographic catchment area. Existing coordination systems between IP organizations needed to be recalibrated to make referral systems functional and harmonize monitoring and evaluation. Insights from early implementation of DREAMS in South Africa and Zimbabwe highlight similar dynamics between IP organizations that struggled with the coordination required for multisectoral programming.^37^ Future multisectoral interventions should invest early in partner coordination systems and allocate resources (i.e., personnel, time, and money) to establish the requisite infrastructure, from electronic data systems to recruitment workplans, for highly synchronized implementation across a consortium of partners.

From the program participant perspective, individual DREAMS interventions appealed to different groups of AGYW and were, therefore, perceived to have varying degrees of impact. For example, younger AGYW valued the safe spaces sessions and older AGYW gravitated toward skill-based, financially oriented interventions (i.e., entrepreneurship training and savings groups). Given program design features (e.g., safe spaces were envisioned as a foundational component of DREAMS in which all AGYW should participate) and variable investment in individual interventions (e.g., lack of capital to support seed funding for microenterprises), enrolled AGYW were not always motivated to participate in available programming. Low uptake and retention in safe spaces programming particularly among older AGYW, who in other DREAMS contexts have also reported navigating competing childcare and employment demands, suggest existing DREAMS programming may require commitments that are infeasible for some AGYW.^37^

Out-of-school AGYW tend to have among the highest HIV risk and, therefore, stand to benefit the most from DREAMS programming; however, recruitment remains an outstanding challenge for DREAMS IP organizations in Zambia and elsewhere. Among out-of-school Zambian girls participating in DREAMS, only half reported characteristics of high HIV vulnerability (e.g., orphanhood, low socioeconomic status, and low comprehensive HIV knowledge).^24^ These more vulnerable AGYW are also more likely to report HIV risks, including early sexual debut and transactional sex.^24^ Consistent with findings from DREAMS evaluations in Kenya and South Africa,^38^^,^^39^ the low prevalence of HIV risk behaviors among DREAMS participants in Zambia could reflect challenges recruiting the highest-risk AGYW, such as younger female sex workers^40^^,^^41^ and married women with children.^37^ Although recruitment strategies have evolved over time, Zambian IP organizations initially met ambitious DREAMS enrollment targets without necessarily reaching the highest-risk AGYW through a school-based enrollment approach. While new evidence from Lesotho and Malawi indicate DREAMS may enhance psychosocial, financial, and other protective assets of these lower-risk AGYW,^42^^,^^43^ substantial differences in sexual risk characteristics between DREAMS participants and unenrolled AGYW suggest convenience recruitment strategies, like school-based enrollment approaches, could attenuate potential effects of DREAMS on key indicators, like HIV incidence, reported in observational studies.^44^^,^^45^ Innovative hotspot-mapping approaches, like those used to identify where young women sell sex in Zimbabwe^40^ and where AGYW meet male partners in Malawi,^46^ are feasible strategies for venue enumeration and targeted recruitment of higher-risk AGYW.

Interventions targeting secondary program audiences, specifically AGYW’s male partners and parents, were plagued by recruitment challenges and suboptimal attendance. In Zambia, parenting and caregiver programs were characterized as among the most resource-intensive and challenging individual interventions in terms of recruitment and sustainability. Population-based surveys in Kenya and South Africa have similarly reported low uptake of caregiving and community-based DREAMS interventions among male partners and parents.^38^ Experiences of Stepping Stones in South Africa^47^^,^^48^ and conditional cash transfer programs^49^^,^^50^ demonstrate the pitfalls of girl and women-focused programming in reducing HIV incidence in AGYW: without direct, prolonged engagement and meaningful involvement of relevant stakeholders (i.e., male partners and parents), achieving the DREAMS Partnership’s primary HIV prevention objectives will be challenging. As parenting programs, like Families Matter! in Zambia, are vital to addressing structural drivers of HIV risk among AGYW, these programs must be backed with sustained financial commitments from donors to appropriately subsidize mobilization, sensitization, and recruitment activities necessary for their success. Likewise, following the advice of DREAMS implementers in South Africa,^51^ eliciting male partners’ service delivery preferences and carefully examining their needs and values related to HIV prevention is essential for recruiting and retaining male partners in DREAMS interventions.

Without direct, prolonged engagement and meaningful involvement of male partners and parents, achieving the DREAMS Partnership’s primary HIV prevention objectives will be challenging.

These findings highlight critical successes and shortcomings of a novel multisectoral approach to delivering complementary HIV prevention services to AGYW in Zambia.

### Limitations

These results, nonetheless, should be considered in light of a few study limitations. First, study recruitment was limited to only 2 urban districts. Although this sample was sufficient for reaching saturation across numerous salient themes identified, insights gleaned from interviews with IP staff and AGYW may be inconsistent with broader patterns and experiences of DREAMS implementation in other settings, where the composition of DREAMS participants and IP staff may be different (e.g., more rural). Second, in the absence of other qualitative data collection methods (e.g., focus group discussions, document review of key DREAMS protocols, and administrative tools), only interviews were used to generate insights into DREAMS implementation, and these may not have elicited specific perspectives or phenomena other data collection methods could have uncovered. Third, the majority of interviewed AGYW were aged 19 years or younger and were in school. The perspectives of in-school AGYW included in this study may, therefore, not map onto the experiences of out-of-school AGYW, who were underrepresented in this study and may have competing values and preferences related to DREAMS implementation. Fourth, this study did not sample secondary program audiences, including AGYW’s male partners and parents or adolescent health care providers, who might have enriched study findings with alternative perspectives on DREAMS implementation. Lastly, the study was conducted approximately 2 years after DREAMS introduction in Zambia, potentially limiting the scope of enabling and constraining factors to DREAMS implementation identified. Future studies of the DREAMS Partnership should be conducted further along the program lifecycle to examine implementation challenges and opportunities that may only emerge several years after program rollout.

## CONCLUSION

The present study’s qualitative approach elicited nuanced perspectives of and experiences with DREAMS implementation in Zambia, uncovering noteworthy enablers and constraints to DREAMS rollout and future scale-up. To reach ambitious program coverage targets without deprioritizing fidelity and effectiveness, these findings underscore the importance of early investment in coordination infrastructure, agile implementation workplans, and resource mobilization for transition planning. As the South African experience with the DREAMS Partnership has demonstrated, emphasizing lofty enrollment targets over recruitment of the highest-risk AGYW who stand to benefit the most from DREAMS participation can dissuade program acceptability by prospective participants.^51^ Centering the voices of AGYW in the design of DREAMS programming and engaging IP staff more proactively in the target-setting process can help secure buy-in from AGYW and support IP staff in meeting ambitious, albeit realistic, recruitment goals. As emerging evidence shows, DREAMS has not accelerated HIV incidence declines in Kenya or South Africa,^44^^,^^45^ casting doubt on the effectiveness of the DREAMS Partnership’s approach. Donors should increase their financial commitments to DREAMS, as our study emphasizes that implementation gaps—rather than the DREAMS model itself—may be to blame for discouraging evaluation findings reported elsewhere. Given the burgeoning interest in implementation science research of combination HIV prevention programs, findings from this study will become increasingly relevant to program managers, donors, and policy makers tasked with tackling HIV incidence in AGYW using novel, multisectoral approaches guiding programming like DREAMS.

## Notes

**Peer Reviewed**

**First published online:** October 6 2022.
